# Association Between Metabolic Syndrome and Breast Cancer Risk: An Updated Meta-Analysis of Follow-Up Studies

**DOI:** 10.3389/fonc.2019.01290

**Published:** 2019-11-22

**Authors:** Meng Guo, Tingting Liu, Peiting Li, Tianying Wang, Chen Zeng, Meng Yang, Gang Li, Jiang Han, Wei Wu, Ruopeng Zhang

**Affiliations:** ^1^Department of Surgery of Mammary Gland and Thyroid Gland, Jining No.1 People's Hospital, Jining Medical University, Jining, China; ^2^Department of Breast Surgery, Tai'an Central Hospital, Tai'an, China; ^3^Department of Breast Thyroid Surgery, The Third Xiangya Hospital of Central South University, Changsha, China; ^4^Department of Reproductive Medicine, Institute of Reproductive Medicine, The First Affiliated Hospital of Dali University, Dali, China

**Keywords:** metabolic syndrome, breast cancer, incidence, postmenopausal, meta-analysis

## Abstract

**Background:** Association between metabolic syndrome (MetS) and incidence of breast cancer remains to be validated. Moreover, whether menopausal status of the women affects this association is unclear. A meta-analysis was performed to summarize the association between MetS and breast cancer risk.

**Methods:** Follow-up studies were identified by search of PubMed and Embase databases published until May 26, 2019. A random-effect model or fixed-effect model was applied to pool the results according to the heterogeneity. Subgroup analyses according to the menopausal status, ethnic groups, cancer histopathological features, and study design characteristics.

**Results:** Overall, 17 follow-up studies with 602,195 women and 15,945 cases of breast cancer were included. Results of meta-analysis showed that MetS defined by the revised National Cholesterol Education Program's Adults Treatment Panel III criteria was associated with significantly increased risk for breast cancer incidence (adjusted risk ratio [RR] = 1.15, *p* = 0.003). Subgroup analyses showed that MetS was associated with significantly increased risk of breast cancer in postmenopausal women (adjusted RR = 1.25, *p* < 0.001), but significantly reduced breast cancer risk in premenopausal women (adjusted RR = 0.82, *p* < 0.001). Further analyses showed that the association between MetS and increased risk of breast cancer were mainly evidenced from studies including Caucasian and Asian women, reporting invasive breast cancer, and of retrospective design.

**Conclusions:** Menopausal status may affect the association between MetS and breast cancer incidence. Postmenopausal women with Mets are associated with increased risk of breast cancer.

## Introduction

Breast cancer is one of the most common malignancies in women globally, with ~1.4 million new cases diagnosed annually (1, 2). Despite of advances in the treatment strategies in recent decades (3, 4), the mortality of women with invasive breast cancer remains high (1, 2). However, strategies for the primary prevention of breast cancer, particularly targeting the modifiable metabolic factors, remain to be determined, probably because of the inconsistencies regarding the role of metabolic factors in the pathogenesis of breast cancer (5–7). Metabolic syndrome (MetS), defined as a cluster of metabolic abnormalities including abdominal adiposity, insulin resistance, hypertension, and dyslipidemia (8–10), has been related to increased risk of a variety of cancer (11–14). Pathologically, patients with MetS are characterized of chronic inflammation and oxidative stress, which have been involved in the carcinogenesis (13, 15). However, results of epidemiological studies evaluating the association between MetS and breast cancer showed inconsistent results (16–32). Early meta-analyses in 2013 and 2014 showed that MetS may be a risk factor for breast cancer, particularly in postmenopausal women (33, 34). However, only nine observational studies were available at that time, and these meta-analyses also included case-control studies, which may introduce additional recall or interviewer biases (35). Moreover, the limited number of the included studies prevented further analyses of the potential impact of study characteristics on the outcomes, such as menopausal status, ethnic groups, cancer histopathological features, and study design. Considerable follow-up studies have been published on this topic since the last meta-analysis (22–32). Therefore, we performed an updated meta-analysis to evaluate the association between MetS and breast cancer risk and to determine whether study characteristics such as menopausal status of the women et al. affect this association.

## Methods

The meta-analysis was designed and performed in accordance with the MOOSE (Meta-analysis of Observational Studies in Epidemiology) (36) and Cochrane's Handbook (37) guidelines.

### Literature Searching

Electronic databases of PubMed and Embase were systematically searched using the combination of the following terms: (1) “metabolic syndrome” OR “insulin resistance syndrome” OR “syndrome X”; (2) “cancer” OR “tumor” OR “neoplasm” OR “carcinoma”; and (3) “cohort” OR “prospective” OR “retrospective” OR “nested case-control” OR “follow-up” OR “followed.” We applied this extensive search strategy to avoid missing of potentially related studies. The search was limited to studies published in English. The reference lists of original and review articles were also analyzed manually. The final literature search was performed on May 26, 2019.

### Study Selection

Studies were included if they met the following criteria: (1) published as full-length article in English; (2) designed as longitudinal follow-up studies with the minimal follow-up duration of 1 year; (3) included women without breast cancer at baseline; (4) women with MetS were identified as exposure of interest at baseline; (5) women without MetS at baseline were included as controls; (6) documented the incidence of breast cancer during follow-up; and (7) reported the adjusted risk ratios (RRs, at least adjusted for age) and their corresponding 95% confidence intervals (CIs). Definitions of MetS were consistent with that was applied in the original studies. Reviews, editorials, preclinical studies, and non-cohort studies were excluded.

### Data Extracting and Quality Evaluation

Literature search, data extraction, and study quality assessment were independently performed by two authors according to the predefined inclusion criteria. If inconsistencies occurred, discussion with the corresponding author was suggested to resolve these issues. The following data were extracted: (1) name of the first author, publication year, study location, and study design; (2) characteristics and numbers of the women, ethnic groups, criteria for the diagnosis of MetS, and follow-up period; and (3) number of cases with breast cancer during follow-up, and variables adjusted when presenting the RRs. The quality of each study was evaluated using the Newcastle-Ottawa Scale (NOS) (38). This scale ranges from 1 to 9 stars and judges the quality of each study regarding three aspects: selection of the study groups; the comparability of the groups; and the ascertainment of the outcome of interest.

### Statistical Analyses

The association between MetS and breast cancer incidence was measured by RRs in this study. To stabilize its variance and normalized the distribution, RR data and its corresponding stand error (SE) from each study was logarithmically transformed (37). The Cochrane's Q test was performed to evaluate the heterogeneity among the include cohort studies (37, 39), and the *I*^2^ statistic was also calculated. A significant heterogeneity was considered if *I*^2^ > 50%. A random effect model was used to pool the results if significant heterogeneity was found; otherwise a fixed effect model was applied. Sensitivity analyses, by omitting one study at a time, were performed to evaluate the potential influence of certain study on the outcome of the meta-analysis (40). To evaluate the influences of menopausal status, ethnic groups, cancer histological feature and study design on the outcome, subgroup analyses were performed (41). Potential publication bias was assessed by visual inspection of the symmetry of the funnel plots, complemented with the Egger regression test (42). The RevMan (Version 5.1; Cochrane Collaboration, Oxford, UK) and STATA software were used for the statistics.

## Results

### Literature Search

**Figure 1:**
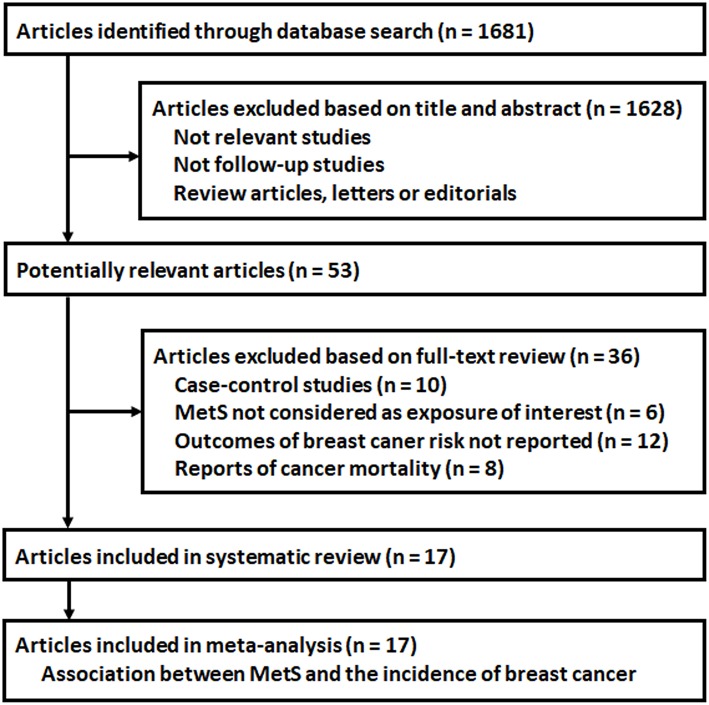
Flowchart of database search and study identification.

The flowchart of database search was shown in Figure 1. Briefly, 1,681 studies were obtained from database search, and 1,628 of them were excluded due to the irrelevance to the objective of the study. For the remaining 53 potential relevant studies that underwent full text review, 36 were further excluded because 10 of them were case-control studies, six did not include MetS as exposure of interest, 12 reported incidences of total cancer or cancers from other sites, and the other eight reported cancer mortality rather than incidence. Finally, 17 follow-up studies were included (16–32).

### Study Characteristics and Quality

Overall, this meta-analysis included 17 follow-up studies (16–32) including 602,195 women and 15,945 cases of breast cancer occurred during follow-up. Since one study provided data by stratification of three age groups, these datasets were included separately (20). The characteristics of the included cohorts were shown in Table 1. Ten of them were prospective cohort studies (16, 17, 20, 22, 24, 25, 27, 28, 30, 32), three were retrospective cohort studies (23, 29, 31), and the other four were nested case-control studies (18, 19, 21, 26). Six of the included studies enrolled postmenopausal women only (18, 19, 21, 24, 30, 31), while the others included both the premenopausal and postmenopausal women, of which five studies provided stratified data by the menopausal status of the women (20, 22, 23, 26, 32). The revised National Cholesterol Education Program's Adults Treatment Panel III (NCEP-ATP III) was used to define MetS for all of the included studies, and for two of the included studies, the International Diabetes Federation (IDF) criteria was also applied (17, 23). Potential confounding factors, including age, education, body mass index, smoking, alcohol drinking, oral contraceptives, hormone therapy, parous/nulliparous, and family history of breast cancer et al. were adjusted to a varying degree in the included studies. The qualities of the included follow-up studies were generally good, with the NOS ranging from seven to nine points.

**Table 1:** Characteristics of the included studies.

**Study**	**Country**	**Design**	**Characteristics of the participants**	**Ethnic groups**	**Number of women**	**Definition of MetS**	**Follow-up period**	**Diagnosis of breast cancer**	**Breast cancer cases**	**Outcome reported**	**Variables adjusted**	**NOS**
							**Years**					
Russo et al. (16)	Italy	PC	Community based women > 40 years	Caucasian	16,677	NCEP-ATP III	1999–2005	Local Cancer Registry	99	Invasive	Age	7
Kabat et al. (18)	the US	NCC	Postmenopausal women	Caucasian	4,396	NCEP-ATP III	1993–1998	Medical chart with pathologic report	165	Total, invasive	Age, education, ethnicity, BMI, oral contraceptive use, hormone therapy, age at menarche age at first birth, age at menopause, alcohol, family history of breast cancer, history of breast biopsy, physical activity, energy intake, and smoking status	8
Inoue et al. (17)	Japan	PC	Community based women > 40 years	Asian	18,176	NCEP-ATP III and IDF	1990–2004	National cancer registries	120	Total	Age, study area, smoking status, alcohol intake, daily total physical activity level, and TC	9
Bjorge et al. (20)	Austria, Norway, and Sweden	PC	Community based women > 40 years	Caucasian	290,000	NCEP-ATP III	1972–2005	National cancer registries	4,862	Total, stratified by three age groups (<50, 50–60, > 60)	Age, study cohort, smoking	8
Agnoli et al. (19)	Italy	NCC	Postmenopausal women	Caucasian	792	NCEP-ATP III	1987–2003	National cancer registries	163	Total	Age, age at menarche, years from menopause, number of full-term pregnancies, age at first birth, oral contraceptive use, hormone therapy use in the past, years of education, family history of breast cancer, breastfeeding, smoking and alcohol consumption	8
Capasso et al. (21)	Italy	NCC	Postmenopausal women	Caucasian	777	NCEP-ATP III	2007–2008	Medical chart with pathologic report	210	Total	Age	7
Bosco et al. (22)	the US	PC	Community based women aged 21–69 years	African	49,172	NCEP-ATP III	1997–2007	National cancer registries	1,228	Total, stratified by menopausal status	Age, education, BMI at age 18, vigorous activity	9
Reeves et al. (24)	the US	PC	Community based women aged > 65 years	Caucasian	8,956	NCEP-ATP III	1988–2002	Medical chart with pathologic report	551	Total, invasive	Age, current hormone use, BMI, and family history of breast cancer	9
Osaki et al. (23)	Japan	RC	General health women	Asian	15,386	NCEP-ATP III and IDF	1992–2007	Tottori prefectural cancer registry	42	Total, including postmenopausal subgroup	Age, smoking status, and alcohol intake	9
van Kruijsdijk et al. (25)	the Netherlands	PC	Patients with vascular diseases	Caucasian	1,589	NCEP-ATP III	1996–2011	National cancer registries	31	Total	Age, smoking status, and alcohol intake	8
Harding et al. (27)	Australia	PC	Community based women	Caucasian	11,031	NCEP-ATP III	1982–2008	National cancer registries	549	Total	Age, smoking, education, and study cohort	9
Agnoli et al. (26)	Italy	NCC	Community based women	Caucasian	1,158	NCEP-ATP III	1993–2008	National cancer registries	593	Total, stratified by menopausal status	Age, parity, age at menarche, smoking status, total physical activity, education, BMI and alcohol consumption	8
Bitzur et al. (28)	Israel	PC	Community based women	Caucasian	6,903	NCEP-ATP III	2000–2010	National cancer registries	186	Total	Age	8
Ko et al. (29)	Korea	RC	Community based population	Asian	37,807	NCEP-ATP III	2002–2013	Local Cancer Registry	359	Total	Age, smoking status, alcohol intake, and exercise	8
Lee et al. (31)	Korea	RC	Women > 50 years for healthy check-up	Asian	23,820	NCEP-ATP III	2002–2013	Local Cancer Registry	131	Total	Age and BMI	8
Kabat et al. (30)	the US	PC	Postmenopausal women	Caucasian	21,000	NCEP-ATP III	1993–2008	Local Cancer Registry	1,176	Invasive	Age, smoking status, smoking, alcohol intake, physical activity, age at first birth, age at menarche, age at menopause, oral contraceptives, hormone therapy, parous/nulliparous, family history of breast cancer, history of breast biopsy, breastfed for more than 6 months, and education	9
Dibaba et al. (32)	the US	PC	National cohort of healthy females	Caucasian or African	94,555	NCEP-ATP III	1995–2011	National cancer registries	5,380	Invasive, stratified by menopausal status and ethnics	Age, BMI, race, physical activity, education, smoking, region, family history of breast cancer, ovary status, current hormonal therapy use, and hysterectomy	9

*NOS, the Newcastle-Ottawa Scale; MetS, metabolic syndrome; NCEP-ATP III, National Cholesterol Education Program's Adults Treatment Panel III; IDF, International Diabetes Federation; PC, prospective cohort; RC, retrospective cohort; NCC, nested case-control; BMI, body mass index; TC, total cholesterol*.

### Association Between the MetS and Breast Cancer Risk

**Figure 2:**
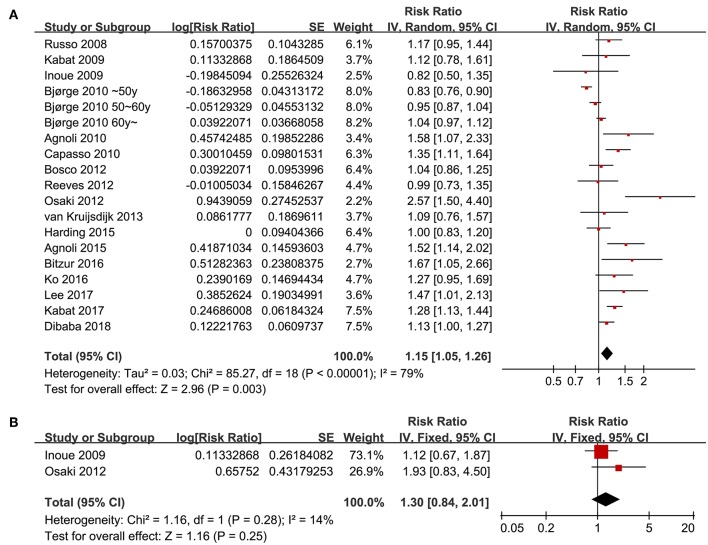
Overall meta-analyses for the association between MetS and breast cancer risk in women. **(A)** Forest plots for the meta-analysis of the association between MetS defined by the revised NCEP-ATP III and breast cancer risk; **(B)** forest plots for the meta-analysis of the association between MetS defined by the IDF criteria and breast cancer risk.

Seventeen follow-up studies (16–32) including 19 datasets were included for the meta-analysis of the association between the revised NCEP-ATP III defined MetS and breast cancer risk. Significant heterogeneity was detected (P for Cochrane's Q test <0.001, *I*^2^ = 79%). Pooled results with a random-effect model showed that women with MetS were associated with significantly increased risk for breast cancer incidence (adjusted RR = 1.15, 95% CI: 1.05 to 1.26, *p* = 0.003; Figure 2A). Results of sensitivity analyses by omitting one study at a time did not significantly change the results (adjusted RR: 1.13 to 1.17, *p* all < 0.05), suggesting the robustness of the finding. Meta-analysis with two studies (17, 23) showed that women with IDF criteria defined MetS were associated with a non-significant increased risk of breast cancer risk during follow-up (adjusted RR = 1.30, 95% CI: 0.84 to 2.01, *p* = 0.25; *I*^2^ = 14%; Figure 2B).

### Results of Subgroup Analyses

**Figure 3:**
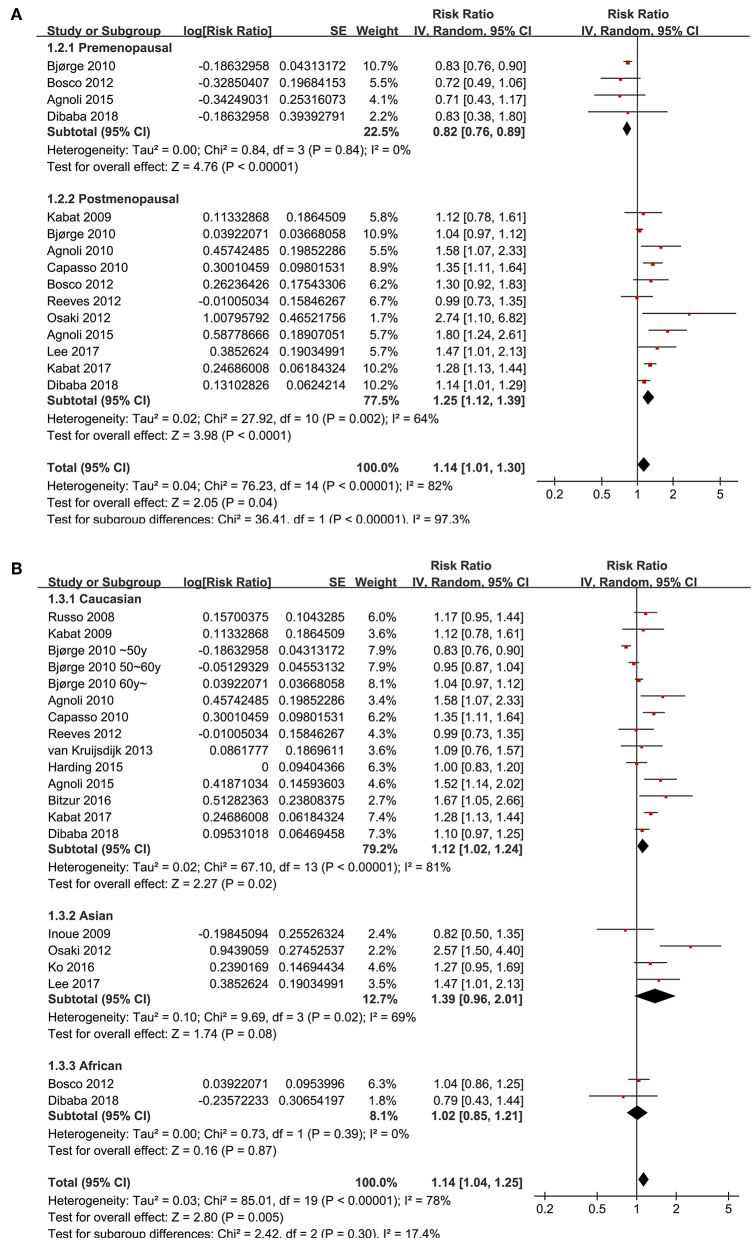
Subgroup analyses for the association between MetS and breast cancer risk in women. **(A)** Stratified by menopausal status; **(B)** stratified by ethnic groups.

**Figure 4:**
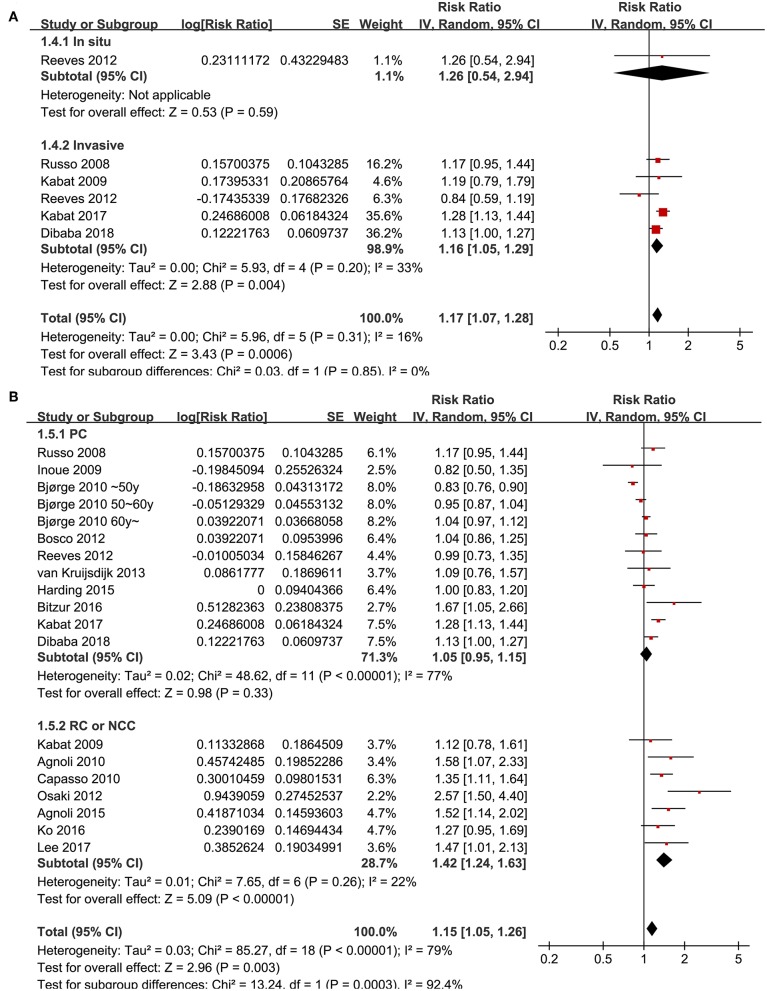
Subgroup analyses for the association between MetS and breast cancer risk in women. **(A)** Stratified by cancer histopathological features; **(B)** stratified by study design characteristics.

Since significant heterogeneity was observed for the studies evaluating the association between the revised NCEP-ATP III defined MetS and breast cancer risk, subgroup analyses was performed to evaluate whether characteristics of menopausal status, ethnic groups, cancer histopathological features, or study design affected the results. Subgroup analyses showed that MetS defined by the revised NCEP-ATP III was associated with significantly increased breast cancer risk in postmenopausal women (11 datasets, adjusted RR = 1.25, 95% CI: 1.12 to 1.39; *p* < 0.001), but with significantly reduced risk of breast cancer in premenopausal women (four datasets, adjusted RR = 0.82, 95% CI: 0.76 to 0.89; *p* < 0.001; Figure 3A). Moreover, MetS was associated with significantly increased risk of breast cancer in Caucasian women (fourteen datasets, adjusted RR = 1.12, 95% CI: 1.02 to 1.24; *p* = 0.02), a trend of increased risk of breast cancer in Asian women (four datasets, adjusted RR = 1.39, 95% CI: 0.96 to 2.01; *p* = 0.08), but unchanged risk of breast cancer in African women (two datasets, adjusted RR = 1.02, 95% CI: 0.85 to 1.21; *p* = 0.87; Figure 3B). In addition, Mets was associated with significantly increased invasive breast cancer (five datasets, adjusted RR = 1.16, 95% CI: 1.05 to 1.29; *p* = 0.004), but the association was not significant for breast cancer *in situ* (one datasets, adjusted RR = 1.26, 95% CI: 1.05 to 2.949; *p* = 0.59; Figure 4A). We also found that the association between MetS and increased breast cancer risk was significant in retrospective studies (seven datasets, adjusted RR = 1.42, 95% CI: 1.24 to 1.63, *p* < 0.001), but not significant in prospective cohort studies (12 datasets, adjusted RR = 1.05, 95% CI: 0.95 to 1.15, *p* = 0.33; Figure 4B).

### Publication Bias

**Figure 5:**
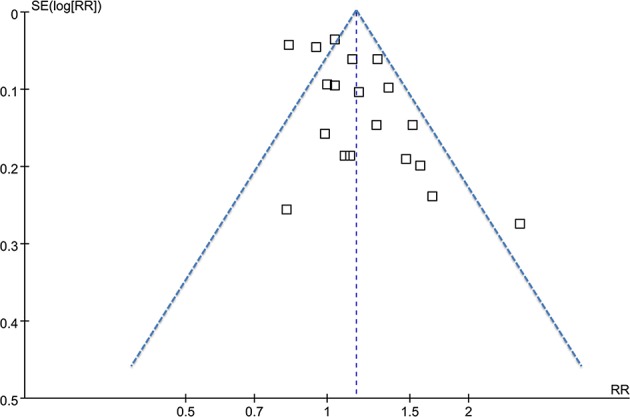
Funnel plots for the meta-analysis of the association between MetS defined by the revised NCEP-ATP III and breast cancer incidence.

The funnel plots for the association between MetS diagnosed by the revised NCEP-ATP III and breast cancer risk were symmetry on visual inspection (Figure 5), suggesting low risk of publication bias. Results of Egger's regression test also showed similar results (*p* = 0.422). Publication bias for the meta-analysis of IDF defined MetS and breast cancer risk was difficult to estimate since only two studies were included.

## Discussion

In this meta-analysis of longitudinal follow-up studies, we found that women with MetS were associated with significantly increased incidence of breast cancer during follow-up. Interestingly, stratified analyses showed that MetS was associated with increased risk of breast cancer in postmenopausal women, but with reduced risk of breast cancer in premenopausal women. Stratified analyses also showed that the significant association between MetS and breast risk was mainly driven by studies including Caucasian and Asian women, reporting outcomes of invasive breast cancer, and those of retrospective design. Taken together, results of our study confirmed previous findings that MetS is associated with significantly increased risk of breast cancer in postmenopausal women. Moreover, results of our study suggested that the menopausal status of the women may be an important modifier for the association between MetS and breast cancer risk. Premenopausal women with MetS may be associated with reduced risk of breast cancer.

Our study has a few strengths compared to previous meta-analyses of the same topic. Firstly, we included longitudinal follow-up studies only, and case-control studies were excluded from the meta-analysis. This is to control the potential recall and interviewer biases inherited in case-control studies. Secondly, we extracted the most adequately adjusted RRs to reflect a potentially independent association between MetS and breast cancer risk. Thirdly, the numbers of included follow-up studies were relatively large (nineteen datasets from 17 follow-up studies) compared with those previous published meta-analyses (nine observational studies including case-control studies), which allowed us to analyze the potential study characteristics on the association between MetS and breast cancer incidence. Finally, our subgroup analyses for the first time showed that menopausal status of the women may be an important modifier for the association between MetS and breast cancer risk, and premenopausal women with MetS may be associated with reduced risk of breast cancer.

The potential mechanisms underlying the independent association between MetS and increased risk of breast cancer in postmenopausal women are likely to be multifactorial. Pervious experimental studies showed that insulin resistance and chronic inflammation are the characterized pathophysiological features in MetS people (13). Insulin resistance could lead to compensatory hyperinsulinemia, which enhanced the cross-binding of insulin to the insulin-like growth factor-1 (IGF-1) receptors expressed on breast epithelial cells (43). The activated IGF-1 pathway may stimulate the carcinogenesis of the breast (43). Moreover, hyperinsulinemia may also accelerate the pathogenesis of breast cancer by stimulation of hepatic IGF-1 synthesis and inhibition the hepatic expression of IGF-1 receptors, leading to an increased circulating IGF-1 level (43). Also, the chronic low-grade inflammation in MetS patients has also been involved in the development of many malignancies, including breast cancer (44). A previous study in obesity-resistant BALB/c strain of female mice showed that a high-fat diet could stimulate growth of an estrogen receptor (ER) -negative murine mammary carcinoma cell line, and its metastasis from the orthotropic injection site to the lungs and liver. This accelerated cancer progression was accompanied by enhanced tumor-related angiogenesis and increased serum concentrations of several proinflammatory cytokines, including interleukin 6, and leptin, which suggested the potential association between MetS, inflammation, and carcinogenesis (45). Moreover, in women with breast cancer, inflammation in the tumor microenvironment, with local elevation in the expression of proinflammatory cytokines (such as tumor necrosis factor-α), has also associated with increased invasiveness and a poor prognosis (46). Although all of the components of MetS have been linked with an increased risk of breast cancer in postmenopausal women in a previous meta-analysis, the combination of these components in MetS seemed to confer stronger association than individual components (33). The key mechanisms and the exact molecular signaling pathways that underling the association between MetS and increased breast cancer risk in postmenopausal women deserve further investigation.

Interestingly, results of subgroup analyses showed that menopausal status of the women may modify the association between MetS and breast cancer incidence, and unlike postmenopausal women, MetS may be associated with reduced risk of breast cancer in premenopausal women. The potential reasons for the diverse associations between MetS and breast cancer risk in premenopausal and postmenopausal women remain unclear. However, these findings were consistent with previous observations that evaluating the association between obesity, diabetes and breast cancer risk. In an early meta-analysis evaluating body mass index (BMI) and breast cancer risk, the authors found that every 5 kg/m^2^ increase of BMI was associated with 12% increased risk of breast cancer in postmenopausal women, but 8% reduced risk in premenopausal women (47). Similarly, another meta-analysis showed that type 2 diabetes mellitus (T2DM) was associated with 16% increased risk of breast cancer in postmenopausal women (48), but 9% reduced risk in premenopausal women, which was also validated in an updated meta-analysis (49). The diverse of association between MetS and breast cancer risk according to the menopausal status could be partly explained by the potential differences for the clinical and histopathological features in premenopausal and postmenopausal women. For example, studies showed that obesity is associated with lower risk of hormone receptor-positive breast cancer, but higher risk of hormone receptor-positive negative cancer in premenopausal women; while obesity is associated with higher risk of hormone receptor-positive breast cancer in postmenopausal women. The associations between MetS and different subtypes of breast cancer according to the menopausal status of the women should be validated in future studies. Our subgroup analyses also showed that the association between MetS and increased risk of breast cancer were mainly evidenced from studies including Caucasian and Asian women, and those reporting invasive breast cancers. For the association between MetS and risk of breast cancer in Black women, and the association between MetS and breast cancer *in situ*, further studies are needed because the datasets available for these subgroups were too few to come to a firmed conclusion. In addition, subgroup analysis also indicated that MetS was not associated with significantly affected risk of breast cancer in prospective cohort studies, but a higher risk of breast cancer in MetS women was observed in studies with a retrospective design. Since retrospective studies are likely to be confounded by recall biases (35), results of meta-analysis with prospective cohort studies are more reliable. This may reflect the finding of a previous meta-analysis which included substantial number of studies with retrospective or cross-sectional design and showed that MetS was associated with higher risk of breast cancer (34).

Our study has limitations, which should be considered when interpreting the results. Firstly, as a nature of meta-analysis of observational studies, we could not exclude other residual confounding factors that may contribute to the association between MetS and breast cancer risk, such as treatments with metformin (50). Secondly, although we analyzed MetS by revised NCEP-ATP III or IDF criteria separately, association between MetS defined by other criteria and breast risk should also be explore. Thirdly, a causative relationship between MetS and breast cancer pathogenesis could not be retrieved based on our study since it was a meta-analysis of observational studies. Fourthly, results of some subgroup analyses (such as those in black women or for the outcome of breast cancer *in situ*) should be confirmed in future studies since limited datasets were available. Finally, as mentioned above, breast cancer is a heterogeneous disease with different histopathological features. The association between MetS and different subtypes of breast cancer should be evaluated in the future.

In conclusion, our updated meta-analysis showed that MetS is associated with significantly increased risk of breast cancer in postmenopausal women. Moreover, menopausal status of the women may be an important modifier for the association between MetS and breast cancer risk, and premenopausal women with MetS may be associated with reduced risk of breast cancer.

## Author Contributions

PL and WW designed the study. PL, TW, and CZ performed database search, data extraction, and quality evaluation. PL, MY, GL, and JH performed data analyses. PL and WW drafted the manuscript. All authors critically revised the manuscript and agreed submission.

### Conflict of Interest

The authors declare that the research was conducted in the absence of any commercial or financial relationships that could be construed as a potential conflict of interest.
